# Florence “blues” are clothed in triple basic terms

**DOI:** 10.1177/20416695221124964

**Published:** 2022-10-03

**Authors:** Maria Michela Del Viva, Ilaria Mariani, Carmen De Caro, Galina V. Paramei

**Affiliations:** NEUROFARBA, Università degli Studi di Firenze, Florence, Italy; Department of Psychology, Liverpool Hope University, Liverpool, UK

**Keywords:** Italian blue basic terms, Florence, Tuscany, BLUE area color naming, CIELAB

## Abstract

Psycholinguistic studies provide evidence that Italian has more than one basic color term (BCT) for “blue”: consensually, *blu* denotes “dark blue,” while “light-and-medium blue,” with diatopic variation, is termed either *azzurro* or *celeste*. For Tuscan speakers (predominantly from Florence), the BLUE area is argued to linguistically differentiate between *azzurro* “medium blue” and *celeste* “light blue.” We scrutinized “basicness” of the three terms. Participants (*N* = 31; university students/graduates born in Tuscany) named each chip of eight Munsell charts encompassing the BLUE area (5BG-5PB; *N* = 237) using an unconstrained color-naming method. They then indicated the “best exemplar” (focal color) of *blu, azzurro* and *celeste*. We found that frequencies of the three terms and of term derivatives were comparable. Referential meaning of *blu*, *azzurro*, and *celeste* was estimated in CIELAB space as L*a*b*-coordinates of the mean of focal colors and as “modal” categories, that is, dispersion around the mean. The three “blue” terms were distinct on both measures and separated along all three CIELAB dimensions but predominantly along the L*-dimension. Our results provide evidence that Tuscan speakers require all three terms for naming the BLUE area, categorically refined along the lightness dimension. Furthermore, *celeste* appears to be a third BCT for “blue,” along with commonly considered BCTs *azzurro* and *blu*. The “triple blues” as BCTs for Tuscan speakers are in contrast with outcomes of two “blue” basic terms estimated by using the same methodology in two other locations in Italy—*azzurro* and *blu* (Verona, Veneto region) or *celeste* and *blu* (Alghero, Sardinia).

## Introduction

According to Berlin and Kay (1969/1991, p. 6), a color term is defined as basic if it is (a) monolexemic, (b) not subsumed under the meaning of any other basic color term (BCT), (c) applied not to a limited class of objects, and (d) psychologically salient for all informants. According to the Berlin and Kay hypothesis, languages with developed color lexicon have maximally 11 BCTs. This was reiterated more recently: “although different languages encode in their vocabularies different numbers of basic color categories, a total inventory of exactly 11 basic color categories exists from which the 11 or fewer basic color categories of any language are always drawn” (Kay, 2015, p. 245).

Novel basic color categories (BCCs) were demonstrated to emerge due to needs for communication efficiency (Gibson et al., 2017; Jameson & Komarova, 2009; Zaslavsky et al., 2019, 2022). Other factors drive the enrichment of the color lexicon, too, such as salient features of the environment and cultural complexity (Josserand et al., 2021). By analyzing, specifically, the emergence of “blue” category/term, the authors found out that it arises from presence of large water bodies (like a sea) and visibility of a blue sky, as well as in those societies where complex blue dyeing techniques are used to color artefacts.

Notably, the tenet of the BCT upper limit has been questioned in last years: it was demonstrated that several languages around the world require more than one BCT to denote specific areas of color space that, in the Berlin and Kay model, are named by one BCT. Relevant to the present study is categorical partition of BLUE area of color space: in a number of languages it requires two BCTs that differentiate light and dark(er) shades of blue. The classic example is Russian *sinij* “dark blue” and *goluboj* “light blue,” with the latter named by Berlin and Kay (1969/1991) as a potential 12^th^ BCT. Recent reviews of linguistic and psycholinguistic studies of the two “Russian blues” underscored Berlin and Kay’s conjecture (Martinovic et al., 2020; Paramei, 2005, 2007). Furthermore, two “blues” are recorded in some other Slavic languages (Belarusian, Polish, and Ukrainian), several circum-Mediterranean languages (Greek, Maltese, and Turkish), some Spanish dialects in Latin America, and two Far East languages (Japanese and Thai) (for a review, see Paramei & Bimler, 2021). The additional “blue” category, as argued by Paramei (2005), is “culturally basic” providing evidence in favor of the weak relativity hypothesis, that is, an impact of language on perception (Malt & Majid, 2013).

The “BLUE challenge” also encompasses Italian. According to Italian linguists, Italian BCT for “blue” is *azzurro* (Giacalone Ramat, 1978; Grossmann, 1988; Mazzoni & Grossmann, 1988). However, linguistic studies provided evidence that, along with *azzurro*, also *blu* and *celeste* are salient terms in both spoken language (Giacalone Ramat, 1978) and written language (Grossmann, 1988) across various Italian dialects suggesting the three terms as BCT candidates*.* Along with these terms, multiple non-BCTs are used for denoting blue color in Italian, such as “old” terms *indaco* and *turchese*/*turchino* or metonymic, culture-specific *carta da zucchero* (e.g., Albertazzi & Da Pos, 2017; Grossmann, 1988; Kristol, 1979; Paggetti et al., 2016; Sandford, 2015; Uusküla & Bimler, 2016)*,* as well as recent terms like *ciano*, *cobalto*, *pervinca*, or *petrolio* (e.g., Albertazzi & Da Pos, 2017; Grossmann & D’Achille, 2016; Uusküla, 2014; Valdegamberi et al., 2015)*.*

*Azzurro* is indeed deeply entrenched in Italian: there is converging evidence from linguistic corpus analysis (Grossmann & D’Achille, 2016; Ronga, 2009), listing task (Bimler & Uusküla, 2018), idiomatic expressions (Ronga et al., 2014; Sandford, 2015), and derivational productivity (Grossmann & D’Achille, 2019; Mazzoni & Grossmann, 1988). *Azzurro* has been attested already in the ninth century, originally having denoted lapis lazuli (Frison & Brun, 2016). According to Kristol (1979) and De Mauro (1983), initially *azzurro* belonged solely to the written language and was absent in Italian dialects; it entered the spoken language after the political unification of Italy during *Risorgimento* (1815–1871), when school education became affordable to general population.

*Celeste* originates from Latin *caeruleus*, derived from *caelu(m)* “sky,” and at the time had no color meaning. With a symbolic religious meaning, but also with a color sense denoting light shades of blue, it is attested in the thirteenth century; in addition, it became associated with fabrics dyed in light blue (termed *cilestro*, *cilestra*). Since then *celeste* has become common in Italian dialects (Giacalone Ramat, 1967; Grossmann, 1988; Grossmann & D’Achille, 2016; Kristol, 1979; Tesoro della Lingua Italiana delle Origini, 1997–).

*Blu* was the last one to enter Italian at the end of the seventeenth century (Grossmann & D’Achille, 2016). It is a loanword derived from an early form of French *bleu* and had been adopted from Germanic languages (Giacalone Ramat, 1967). *Blu* was deployed to lexicalize deep (dark/navy) blue conceivably as a result of its use in the cloth trade (see Pastoureau, 2001, p. 127). Nowadays, it is considered to be the most widespread “blue” term in Italian (Sandford, 2015). A considerable increase in its use during the twentieth century, as argued by Grossmann and D’Achille (2016), is due to the great number of calques and loans caused by globalization implying contact with English, French, and German. As a result, in contemporary Italian, *blu*, a homophone of English *blue*, is frequently used as an umbrella term for describing the whole BLUE area (Grossmann & D’Achille, 2016; Sandford, 2015).

In addition to the linguistic studies overviewed above, recent psycholinguistic studies, carried out in different regions of Italy, provide converging evidence that in Italian more than one BCT is required for naming BLUE area (Bimler & Uusküla, 2014, 2018; Paggetti & Menegaz, 2012; Paggetti et al., 2016; Paramei et al., 2014, 2018; Sandford, 2015; Uusküla, 2014; Uusküla & Bimler, 2016; Valdegamberi et al., 2015). There is controversy, though, of whether BLUE area is denoted by two BCTs (Paggetti & Menegaz, 2012; Paggetti et al., 2016) or three BCTs (Bimler & Uusküla, 2014).

Across all linguistic and psycholinguistic studies, the authors are unanimous that *blu* is the counterpart of English “dark/navy blue.” However, the second Italian BCT is argued to be lexicalized as either *azzurro* (Paggetti et al., 2016; Valdegamberi et al., 2015) or *celeste* (Paramei et al., 2018). Furthermore, in some speakers’ opinion *azzurro* denotes a shade in-between *celeste* “light-blue” and *blu* “dark blue” (Grossmann, 1988), whereas for others *azzurro-*meaning is similar to that of *celeste*, with both being in opposition to *blu* (Albertazzi & Da Pos, 2017; Kristol, 1979).

The degree of use of the three “blue” terms in the spoken language is known to be subject to diatopic variation, that is, a variety of dialects spoken in different regions of Italy (De Mauro, 1983; Grossmann & D’Achille, 2016; Paramei et al., 2018). In particular, for speakers from Verona (Veneto region), *azzurro* is the second BCT denoting the BLUE area and corresponds to “light-and-medium blue” (Paggetti et al., 2016; Paramei et al., 2014, 2018). In comparison, for speakers of Catalan-Algherese dialect (Alghero, Sardinia), the second BCT is *celeste*, which denotes light(er) shades of blue, while *azzurro*, with the meaning of “dark medium blue,” apparently is not a basic (Paramei et al., 2018).

For speakers from Florence (Tuscany), BLUE area seems to be “clothed in triple blues,” with *azzurro* denoting “medium blue” and *celeste* reserved for “light blue” (Bimler & Uusküla, 2014; Uusküla, 2014). It is noteworthy that Bimler and Uusküla (2014) employed the Color-aid Corporation set based on Ostwald’s color solid (https://coloraid.com/). Also, these authors used a much smaller number of chips (*N* = 55), unlike the large number of chips (*N* = 237) from the Munsell Color set employed in the Alghero and Verona studies (Paramei et al., 2014, 2018).

The aim of the present study was to further investigate the basic “blue” term inventory for Tuscan speakers, predominantly from Florence, and possible effects of the stimulus set on estimates of the term basicness. In the context of the present study, we heeded two of Berlin and Kay’s criteria of a BCT. Specifically, we explored whether the meaning of each term is not included in the meaning of one or the other “blue” term (criterion (b)). Also, we assessed psychological salience of each “blue” term (criterion (d))—stability of reference across informants; and occurrence in ideolects of all informants.

The objective was to explore whether the discrepancies found in the number of Italian basic “blue” terms—two in the Alghero and Verona studies (Paramei et al., 2014, 2018) and three in the study of Florence speakers (Bimler & Uusküla, 2014)—resulted from regional dialectal varieties or from the color set used. In other words, we aimed to disentangle diatopic and methodological factors that may have affected the naming of the BLUE area. To this end, color names were elicited from a sample of functionally monolingual Italian participants born and raised in Tuscany, that is, similar as the one tested in Bimler and Uusküla’s (2014) study. Furthermore, all our participants were comparable in age and (university) education to ensure that they were exposed to the same culture, with minimal dialectal variation. To enable direct comparisons with previous results, we adopted the same procedure and stimulus set, as the one employed in the Alghero and Verona studies (Paramei et al., 2014, 2018).

## Methods

### Participants

Participants were Italian monolingual university students or graduates (*N* = 31; 17 females) aged 19–26 y.o.; mean age 22.9 ± 2.1. All participants resided and were born in Tuscany. Further information about participants can be found in Table S1 of the Supplementary Materials. All participants had normal color vision assessed by the Ishihara Pseudoisochromatic Plates (Ishihara, 1987) and normal or corrected-to-normal visual acuity. None had reported any ocular disease, eye surgery, diabetes, or use of a medication that could have affected color vision. The study was conducted according to the guidelines of the Declaration of Helsinki and approved by the local ethics committee (“Commissione per l’Etica della Ricerca,” University of Florence, July 7, 2020, No. 111). All participants gave their written informed consent prior to the participation in the study.

### Stimuli

**Figure 1.**
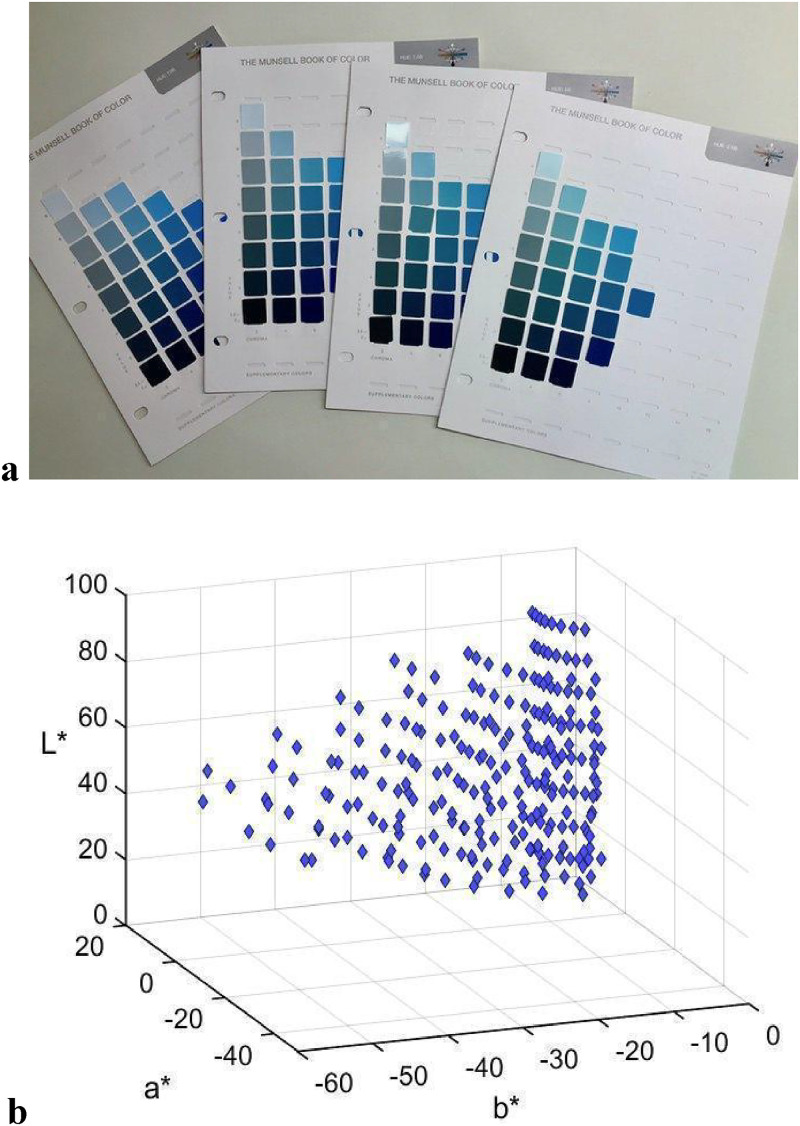
Stimuli (a) examples of three BLUE area Munsell charts. Photo credit: https://helloartsy.com/introduction-to-munsell-color/. Fair use of the image for research and scholarship purposes, as defined by Section 107 of the Copyright Act. (b) Munsell BLUE area stimuli (*N* = 237) presented in CIELAB space.

Eight charts from *The Munsell Book of Color* (glossy edition) were employed. The charts embraced the BLUE area, with Hue 7.5BG, 10BG, 2.5B, 5B, 7.5B, 10B, 2.5PB, and 5PB, as illustrated in Figure 1. Value of the Munsell chips varied between 2 and 9, and Chroma varied (even number notation) from 2−10, or 2–12 in 10B, 2.5PB, 5PB. For purposes of further analysis, Munsell coordinates of the stimuli (*N* = 237) were re-notated in the CIELAB space.

### Procedure

**Figure 2.**
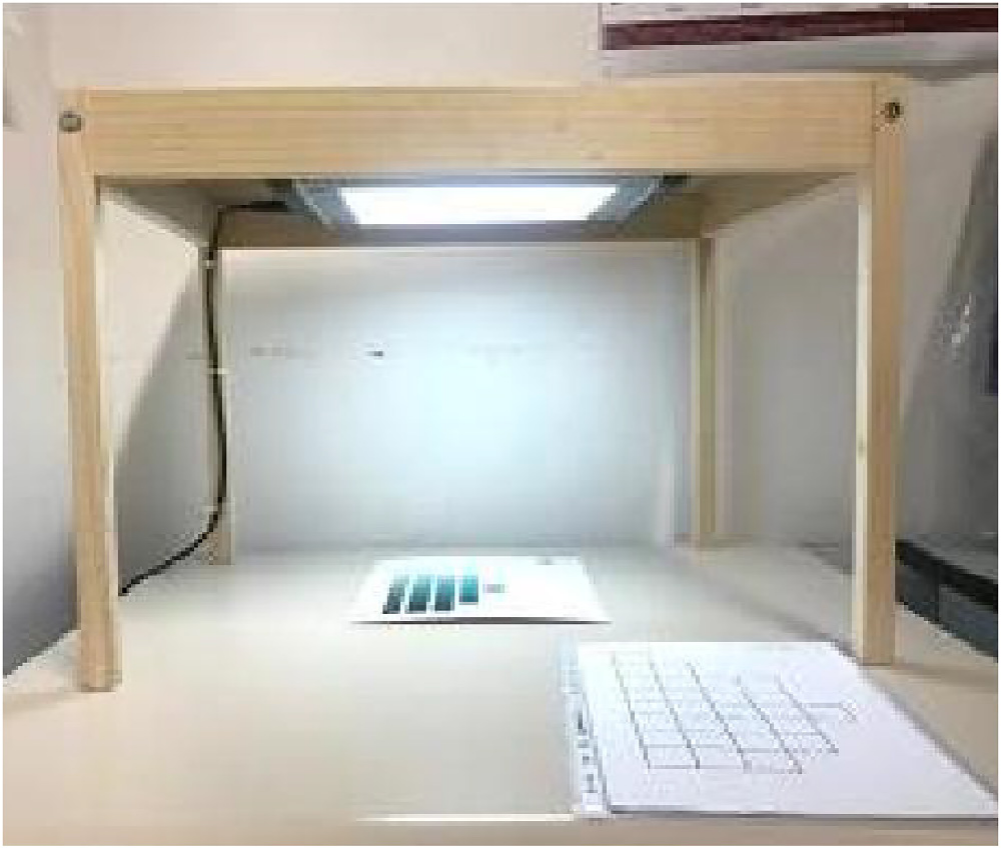
Viewing booth with standardized lighting of Munsell charts and response sheets.

Participants were adapted to mesopic lighting in an otherwise dark room for at least 10 min, the temporal window ensuring dark adaptation of cones (Pirenne, 1962). Following this, the charts were presented in a viewing booth under D65-metameric illumination (Just Normlicht Mini 5000; Fa. Colour Confidence) suspended 40 cm above the chart (Figure 2). At the chart surface, luminance was 220 cd/m^2^ (measured by the PR-650 SpectaScan Colorimeter; Photo Research, Inc.), corresponding to illuminance of 1387 lux.

For naming the Munsell chips, an unconstrained color-naming method was used: participants were requested to name each chip freely. A certain color term could be used by a participant for naming more than one chip. The response sheets consisted of a replica of the structure of the eight Munsell charts. Participants were asked to write color names within blank cells corresponding to the chart chips (Figure 2). Participants worked row by row across the chart from top to bottom. Elicited color names included hue terms (e.g., *celeste, azzurro*, *blu*, *indaco*, *tiffany*), compound terms (e.g., *celeste-lilla, grigio-azzurro*), terms with modifiers (e.g., *blu notte, indaco scuro, turchese chiaro*) or with suffixes (e.g., *bluastro, azzurrognolo*).

Each chart was presented one-by-one in a fixed order, to avoid “the danger that participants tested in one order … will adopt strategies or criteria different from those … adopted by participants tested in a different order” (Mollon et al., 2017, p. 12). Following this, all the participants indicated, across all eight charts shown simultaneously, the “best exemplar” (focal color) of the terms *blu*, *azzurro* and *celeste*. The focal colors were noted by the experimenter on the response sheet and coded by their Munsell Hue, Value and Chroma.

### Data Analysis

Initially, linguistic frequency analysis of all terms offered by all participants was undertaken. Next, raw naming data of all elicited “blue” terms were transformed into their modal correspondents with the focus on *celeste, azzurro,* and *blu*; also, other frequent non-BCTs terms were considered (*turchese*, *indaco*, *cobalto*, *ciano*, etc.).

The modal terms of *celeste*, *azzurro*, and *blu* included the basic forms, as well as their derivatives (e.g., *celestino, azzurrino, bluastro*), compound terms either with an adjective (e.g., *bianco celeste*, *azzurro verde*, *blu nero)* or a noun (e.g., *celeste cielo*, *azzurro acqua*, *blu notte),* and terms with brightness or saturation modifiers (e.g., *celeste intense*, *azzurro scuro*, *blu oltreoceano*) (cf., Bimler & Uusküla, 2014; Grossmann & D’Achille, 2019; Uusküla & Bimler, 2020). Note that non-“blue” terms (*grigio, verde, nero,* etc.), basic in Italian (Paggetti et al., 2016), not exclusive of the BLUE area, were not included in the present analysis.

For psycholinguistic analysis, Munsell coordinates of the stimuli named by the modal terms celest*, azzurr*, and blu* were transformed to CIELAB coordinates (http://www.cis.rit.edu/research/mcsl2/online/munsell.php). For each modal category, the central tendency of the distribution of denotata was computed as the mean of L*, a*, and b* coordinates and standard error of mean along each coordinate. Also, Munsell coordinates of focal colors of *celeste*, *azzurro*, and *blu* were transformed to CIELAB coordinates, and mean and standard error of their distributions were computed.

Normality of distribution of denotata of each of the three modal categories and the focal colors along each CIELAB coordinate was assessed by the Shapiro–Wilk test. Since most datasets were not normally distributed, median and semi-interquartile range (sIQR) values were also computed. Kruskal–Wallis *H* and Mann–Whitney *U* tests were used to assess differences between distributions along each coordinate, across the three categories and pairwise, respectively*.*

Finally, consensus of naming the BLUE area by modal celest*, azzurr*, and blu* was calculated as the percentage of participants (out of 31), who named the same chip with the identical modal term.

## Results

### Most Frequent Terms for the BLUE Area

The tested Tuscan speakers used a large variety of color terms to describe the BLUE area. The total number of different terms is 1,344, while for individual participants, the total number of terms varied between 9 and 153. The list of terms most frequently offered by participants (having occurred in at least 14% cases) is presented in Table 1. Data for each individual participant is reported in Table S2 that lists the elicited color names according to their rank (up to rank 30). In the variety of color descriptors, 42 different combinations were recorded. Each individual participant (apart from one, who did not offer *celeste*) used all three “blue” terms, *celeste*, *azzurro*, and *blu*. Notably, among the three terms, *celeste* has the highest frequency, as is the case for Alghero speakers (Paramei et al., 2014, 2018); it is followed by *azzurro* (rank 5) and *blu* (rank 6). For the participant, who offered only nine color names in total, *celeste*, *azzurro*, and *blu* had ranks 2, 3, and 4, respectively (see Table S2).

**Table 1.** Frequency and Percentage of 78 Color Terms Most Frequently Offered for Naming the BLUE Area by Tuscan Speakers.

Rank	Color Term	Freq.	%	Rank	Color Term	Freq.	%
1	grigio	430	6.11	40	grigio scurissimo	30	0.43
2	grigio scuro	268	3.81	41	grigio verde	28	0.4
3	celeste	263	3.74	42	verde acqua scuro	28	0.4
4	grigio chiaro	236	3.35	43	blu petrolio	27	0.38
5	azzurro	220	3.13	44	grigio molto chiaro	27	0.38
6	blu	179	2.54	45	blu acqua	25	0.36
7	celeste scuro	152	2.16	46	blu opaco	25	0.36
8	blu scuro	148	2.1	47	bianco sporco	24	0.34
9	nero	127	1.8	48	indaco	24	0.34
10	azzurro scuro	116	1.65	49	blu elettrico	23	0.33
11	blu chiaro	113	1.61	50	celeste spento	23	0.33
12	blu notte	83	1.18	51	grigio nero	21	0.3
13	azzurro chiaro	81	1.15	52	grigio bluastro	20	0.28
14	verde acqua	75	1.07	53	blu profondo	19	0.27
15	celeste chiaro	73	1.04	54	blu spento	19	0.27
16	verde scuro	69	0.98	55	grigio celeste	19	0.27
17	celestino	68	0.97	56	turchese	19	0.27
18	azzurro grigio	62	0.88	57	verde bottiglia	19	0.27
19	grigiastro	62	0.88	58	blu sporco	18	0.26
20	grigio topo	55	0.78	59	carta da zucchero	18	0.26
21	grigino	53	0.75	60	grigio fumo	18	0.26
22	grigio azzurro	52	0.74	61	blu cobalto	17	0.24
23	blu grigio	51	0.72	62	bluastro	17	0.24
24	celeste grigio	51	0.72	63	celestino chiaro	17	0.24
25	bianco	46	0.65	64	grigio opaco	17	0.24
26	grigio blu	46	0.65	65	antracite	16	0.23
27	azzurro sporco	44	0.63	66	celeste cielo	16	0.23
28	azzurrino	43	0.61	67	celeste grigio scuro	16	0.23
29	azzurro opaco	43	0.61	68	verde grigio	16	0.23
30	blu grigiastro	41	0.58	69	azzurrino chiaro	15	0.21
31	celeste grigiastro	38	0.54	70	azzurro acqua	15	0.21
32	celeste sporco	36	0.51	71	blu acceso	15	0.21
33	celeste opaco	35	0.5	72	grigio molto scuro	15	0.21
34	azzurro grigiastro	33	0.47	73	verde smeraldo	15	0.21
35	azzurro cielo	31	0.44	74	blu azzurro	14	0.2
36	blu mare	31	0.44	75	blu grigio scuro	14	0.2
37	verde	31	0.44	76	blu scurino	14	0.2
38	celeste acceso	30	0.43	77	grigio tendente nero	14	0.2
39	grigio chiarissimo	30	0.43	78	turchese scuro	14	0.2

Relative occurrences of the three modal terms are different, though: participants offered 326 different combinations containing blu* (e.g., *blu notte*, *bluastro grigio*), 272 descriptors with azzurr* (e.g., *azzurrastro scuro*, *azzurro elettrico*), and 262 terms with celest* (e.g., *celestino pallido*, *celeste fumo*).

**Figure 3.**
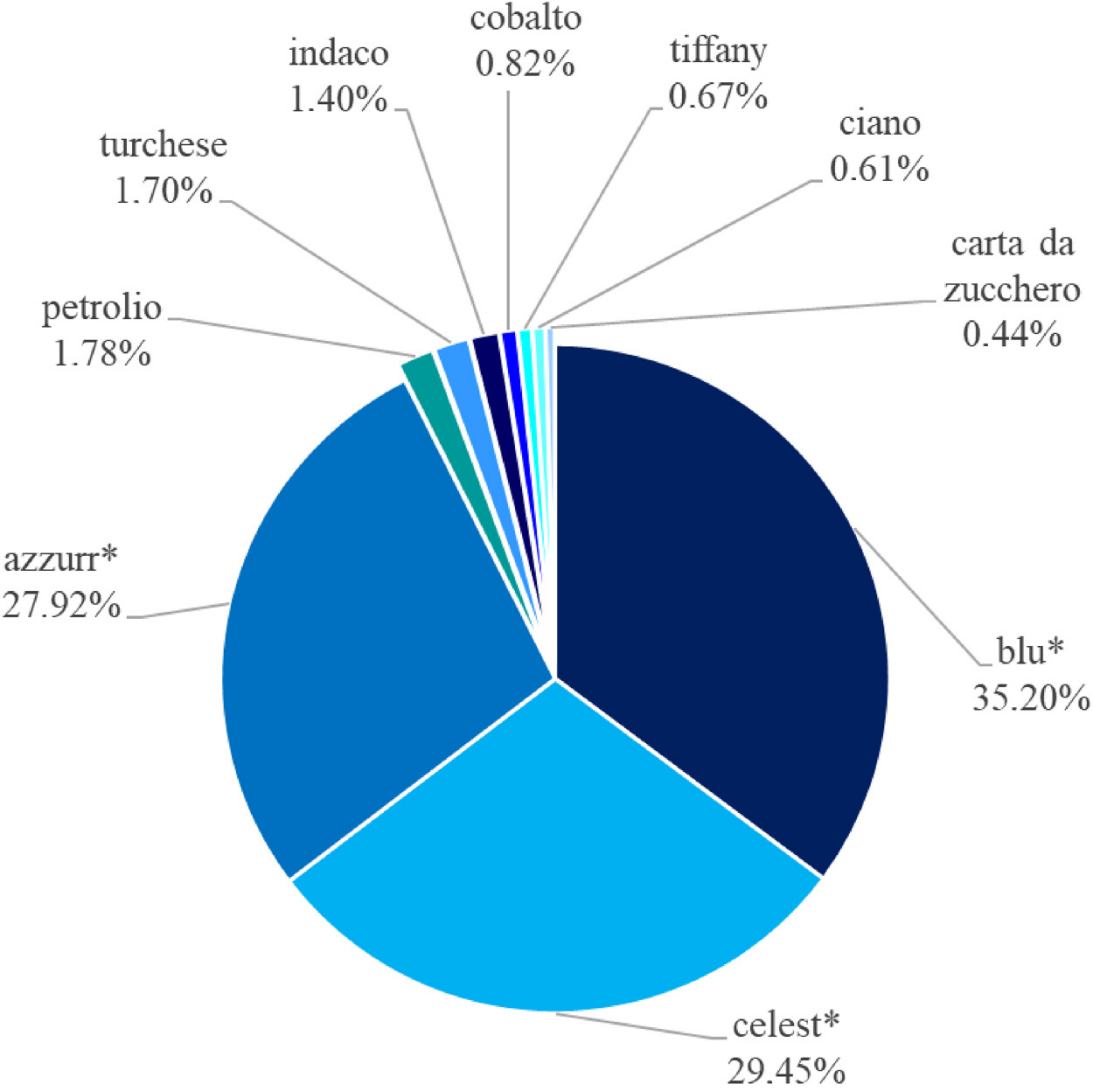
Percentage of modal terms of celest*, azzurr*, and blu* and other Italian non-BCTs frequently used to denote the BLUE area.

Figure 3 shows relative frequencies of use of various “blue” terms denoting BLUE area. Among most frequent non-BCTs, although with low percentages, elicited were *indaco* (rank 48) and *turchese* (rank 56), which, too, were frequently named in previous list and/or naming tasks (Albertazzi & Da Pos, 2017; Bimler & Uusküla, 2018; Grossman & D’Achille, 2016, 2019; Sandford, 2015; Uusküla & Bimler, 2016, Fig. 1(f); Valdegamberi et al., 2015). Relatively frequent in our dataset were, as well, highly entrenched *carta da zucchero* (rank 59) and frequent modern Italian terms like *cobalto* (rank 93), *petrolio* (rank 96), *ciano* (rank 106), and *tiffany* (rank 112), also attested in previous linguistic and psycholinguistic studies (cf., Grossmann & D’Achille, 2016, 2019; Paggetti et al., 2016; Valdegamberi et al., 2015).

Visual inspection of Figure 3 prompts that relative frequencies of modal celest*, azzurr*, and blu* are comparable: 29.45%, 27.92%, and 35.2%, respectively, as are their corresponding absolute frequencies counted across all participants: celest*: 1,539; azzurr*: 1,459; and blu*: 1,839.

### Estimation of Denotata of Modal Terms Celest*, Azzurr*, and Blu*

**Figure 4.**
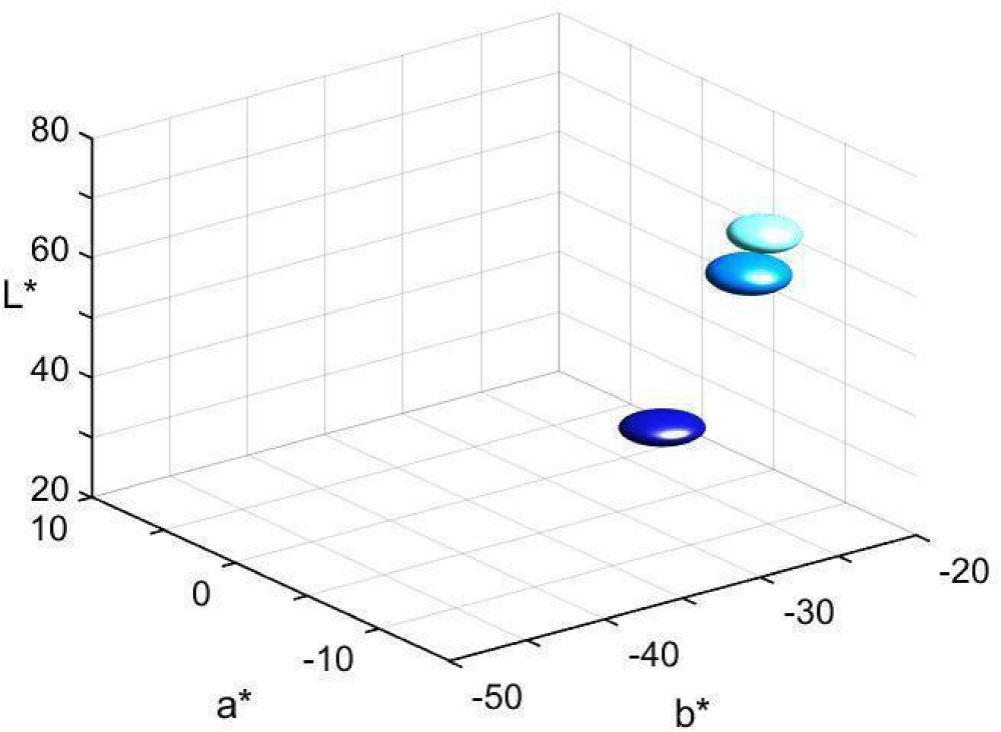
Central tendency of denotative distribution of modal terms celest* (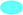), azzurr* (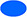), and blu* (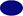)*.* The ellipsoids represent the modal term’s standard error around the mean.

Means and standard errors of the distributions of denotata, in CIELAB space, of the three modal terms, across all participants, are shown in Table 2. (Full denotative distribution of each modal term is shown in Figure S1 of the Supplementary Materials.) Figure 4 shows the three corresponding ellipsoids, whose size represents the reported standard errors around the mean along each of the CIELAB coordinates. The three distributions are quite separated suggesting three different “blue” categories for the employed stimulus set.

**Table 2.** Mean and Standard Error (CIELAB Coordinates) of Ellipsoids of Denotata of Modal Terms Celest*, Azzurr*, and Blu*, and the Corresponding Focal Colors. For Each Term, Indicated is the Number (*N*) of Chips Named so Across all Participants.

	L*		a*		b*	
	Modal	Focal	Modal	Focal	Modal	Focal
*Celeste* (*N* = 1,399)	65.65 (2.42)	70.26 (1.70)	−8.29 (1.73)	−11.56 (1.43)	−23.65 (1.87)	−32.94 (1.80)
*Azzurro* (*N* = 1,329)	61.86 (2.60)	61.87 (2.25)	−9.57 (1.94)	−8.12 (1.16)	−25.84 (2.15)	−40.77 (2.05)
*Blu* (*N* = 1,533)	34.13 (1.93)	30.80 (1.27)	−6.16 (1.84)	5.71 (1.09)	−28.24 (2.23)	−45.25 (0.89)

**Figure 5.**
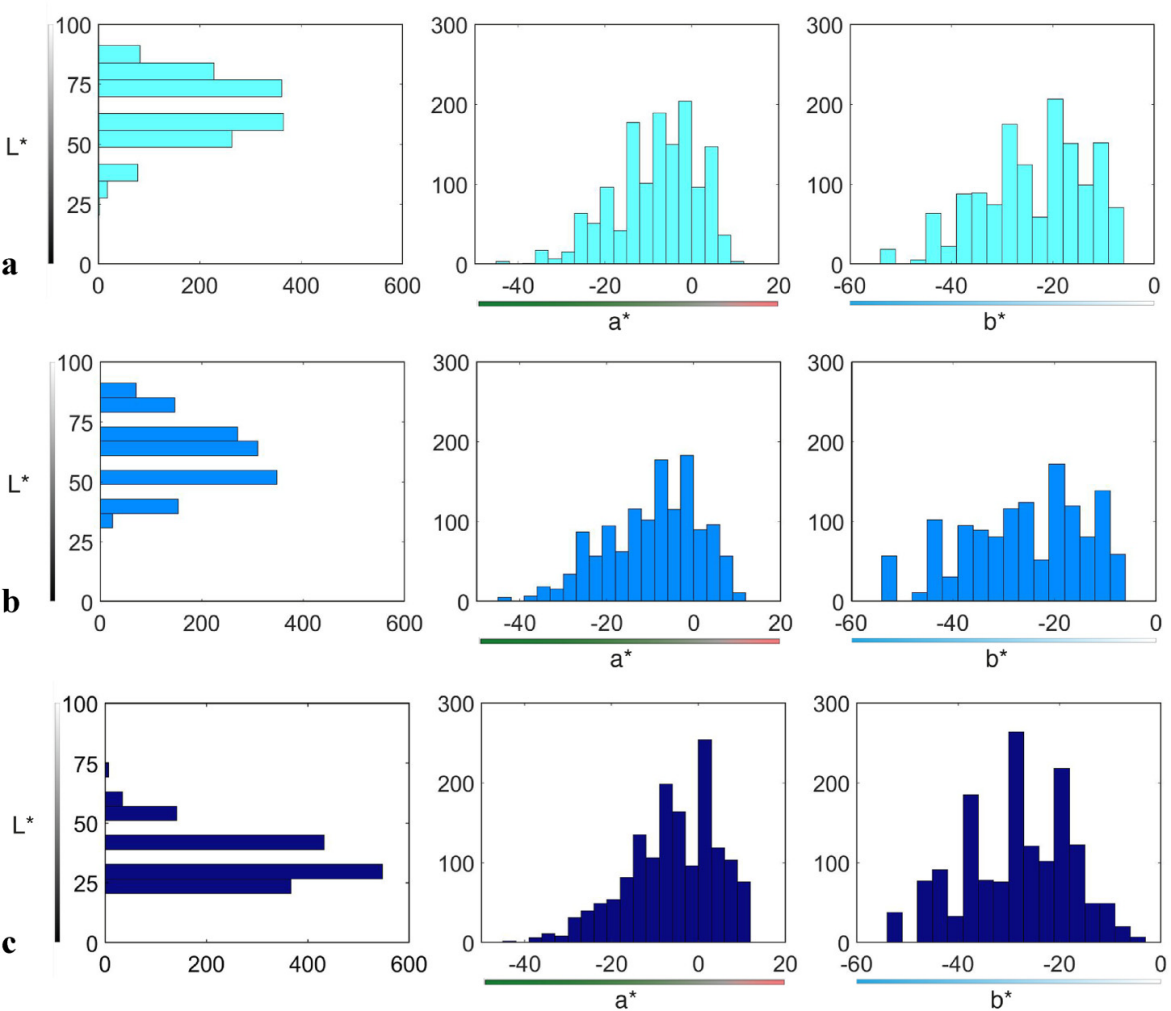
Projections of the distributions of denotata of modal terms celest* (a), azzurr* (b), and blu* (c) along each of the three CIELAB coordinates. Leftmost graphs: *y*-axes are the L*-coordinate of the modal terms; *x*-axes are the cumulative number of chips named by the corresponding modal term. Middle graphs: *x*-axes are the a*-coordinate of the modal terms; *y*-axes are the cumulative number of chips named by the corresponding modal term. Rightmost graphs: *x*-axes are the b*-coordinate of the modal terms; *y*-axes are the cumulative number of chips named by the corresponding modal term.

 Figure 5(a–c) shows distributions of the denotata of modal terms celest*, azzurr*, and blu* separately along each of the L*, a*, and b* coordinates*.* Since none of the L*, a*, or b* distributions met the requirement of normality (*p* < .001 for all datasets), the corresponding medians and sIQRs were calculated and are reported in Table 3.

**Table 3.** Median and Semi-Interquartile Range (CIELAB Coordinates) of Denotata of Modal Terms Celest*, Azzurr*, and Blu*, and the Corresponding Focal Colors. For Each Term, Indicated is the Number (N) of Chips Named so Across all Participants.

	L*		a*		b*	
	Modal	Focal	Modal	Focal	Modal	Focal
*Celeste* (*N* = 1,399)	61.70 (10.01)	71.60 (7.39)	−6.15 (6.90)	−9.70 (5.38)	−22.78 (7.11)	−32.54 (6.48)
*Azzurro* (*N* = 1,329)	61.70 (10.01)	61.70 (8.61)	−8.75 (8.08)	−10.84 (4.83)	−25.41 (9.15)	−44.00 (8.48)
*Blu* (*N* = 1,533)	30.77 (5.22)	30.77 (0)	−5.42 (6.84)	9.36 (3.82)	−27.80 (8.38)	−46.85 (0.18)

The three “blue” categories differ predominantly in their lightness, along the L* coordinate (*χ^2^*(2) = 2,423.77, *p* < .001), as illustrated by Figure 5 (leftmost graphs). In particular, celest* category is lighter than both azzurr* (*U* *=* 1,081,753.50, *z* = 7.444, *p* < .001) and blu* (*U* = 2,052,905.50, *z* = 43.28 *p* < .001), and azzurr* is lighter than blu* (*U* *=* 1,904,582.00, *z* = 40.71, *p* < .001).

The three categories also differ along the a* coordinate (*χ^2^*(2) = 85.77 *p* < .001), as illustrated by Figure 5 (middle graphs): celest* differs from both azzurr* (*U* *=* 985,369.00, *z* = 2.710, *p* < .001) and blu* (*U* *=* 922,367.50, *z* = −6.55, *p* < .001); also azzurr* differs from blu* (*U* *=* 825,480.00, *z* =  − 8.76, *p* < .001), with azzurr* being more “greenish” than the other two terms.

A similar pattern of differences is observed for the b*-coordinate (Figure 5, rightmost graphs), with all three categories being statistically different (*χ^2^*(2) = 156.54 *p* < .001): celest* is different from both azzurr* (*U* *=* 1,081,753.50, *z* = 7.44, *p* < .001) and blu* (*U* = 2,052,905.50, *z* = 43.28, *p* < .001); as are azzurr* and blu* different (*U* *=* 1,904,582.00, *z* = 40.71, *p* < .001). Among the three terms, blu* appears to be of greatest “bluishness.”

### Estimation of Focal Colors of *Celeste*, *Azzurro*, and *Blu* Categories

**Figure 6.**
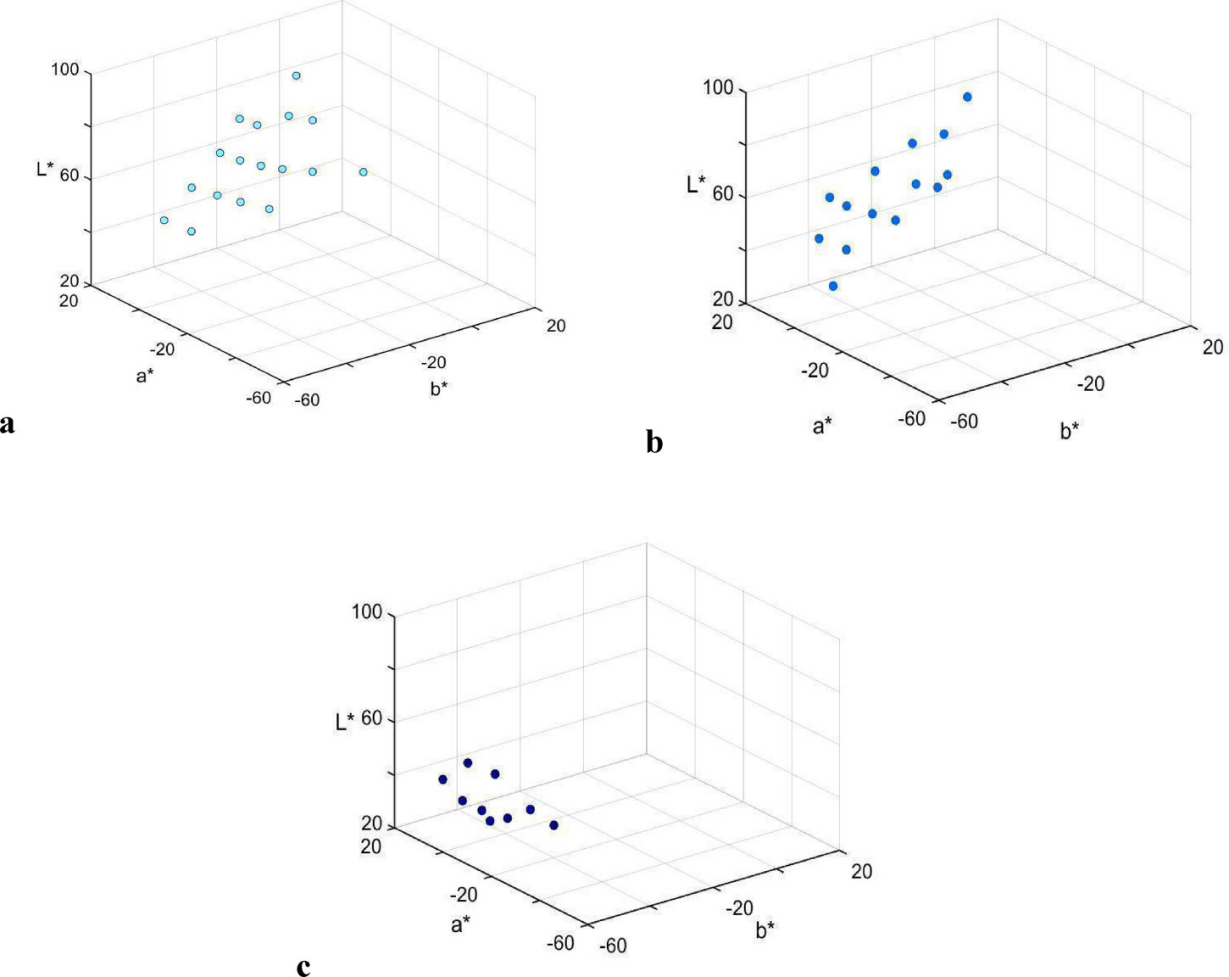
Distribution of denotata of focal colors, across all participants, for (a) *celeste* (17 chips), (b) *azzurro* (13 chips), and (c) *blu* (9 chips) in CIELAB space*.*

**Figure 7.**
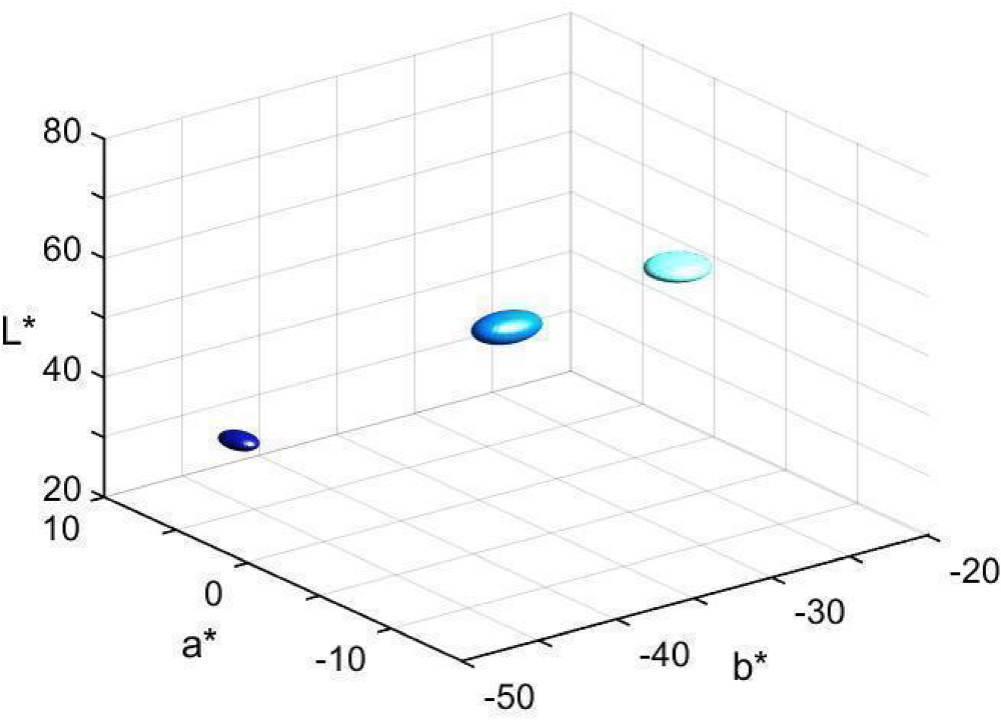
Central tendency of denotative distribution of focal colors of *celeste* (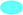), *azzurro* (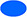), and *blu* (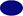)*.* The ellipsoids represent the focal color standard error around the mean.

Distribution of denotata of focal colors of the three categories, across all participants, is shown in Figure 6. The central tendency and dispersion of focal color choices are presented as ellipsoids in Figure 7 (see also Table 2). Note that the dispersion of *blu* foci is smaller than that of the other two terms' foci, the finding addressed below in relation to the naming consensus.

**Figure 8.**
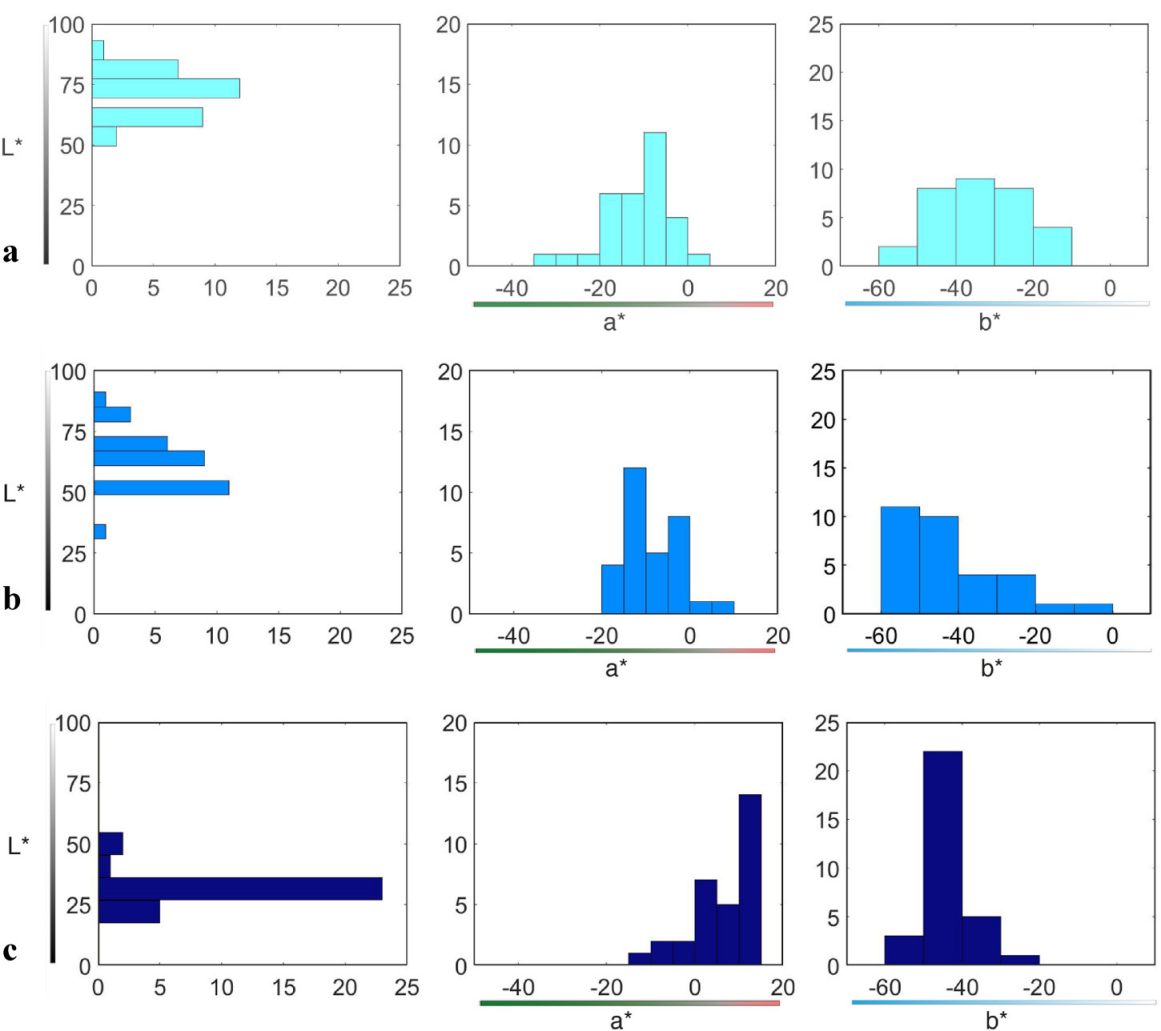
Projections of the distributions of denotata of focal colors of (a) *celeste*, (b) *azzurro*, and (c) *blu* along each of the three CIELAB coordinates. Leftmost graphs: *y*-axes are the L*-coordinate of the chosen focal colors; *x*-axes are the cumulative number of chips chosen as the focal color of the corresponding term. Middle graphs: *x*-axes are the a*-coordinate of the chosen focal colors; *y*-axes are the cumulative number of chosen chips. Rightmost graphs: *x*-axes are the b*-coordinate of the chosen focal colors; *y*-axes are the cumulative number of chosen chips.

Medians and sIQRs of L*, a*, and b* projections of focal colors are presented in Table 3. Kruskal–Wallis *H* test indicated significant differences of the foci of the three categories along the L*-coordinate (*χ^2^*(2) = 2,423.77 *p* < .001), as illustrated by Figure 8 (leftmost graphs). All pairwise comparisons were significant too: *celeste* is lighter than both *azzurro* (*U* = 688.50, *z* = 2.995, *p* < .001) and *blu* (*U* = 959.00, *z* = 6.95, *p* < .001), and *azzurro* is lighter than *blu* (*U* *=* 935.50, *z* = 6.64, *p* < .001). Thus, as expected, the focal color of *celeste* is the lightest, whereas the focal color of *blu* is the darkest.

Differences between focal colors, across the three categories, are also significant for the a*-coordinate (*χ^2^*(2) = 85.77, *p* < .001), as shown in Figure 8 (middle graphs). Pairwise comparisons indicate significant differences between focal colors of *celeste* and *blu* (*U* *=* 38.00, *z* =  − 6.274, *p* < .001), as well as between focal colors of *azzurro* and *blu* (*U* *=* 65.00, *z* =  − 5.904, *p* < .001), while there is no difference in “greenishness” between focal colors of *celeste* and *azzurro* (*U* *=* 389.50, *z* =  − 1.286, *p* = .198).

Finally, there are differences between focal colors of the three categories along the b*-coordinate, too (*χ^2^*(2) = 156.54, *p* <.001), as illustrated by Figure 8 (rightmost graphs). Pairwise comparisons indicate that focal color of *celeste* differs both from *azzurro* (*U* *=* 269.50, *z* =  − 2.98, *p* = .002) and *blu* (*U* *=* 842.00, *z* = 5.13, *p* < .001); in comparison, focal colors of *azzurro* and *blu* are not significantly different (*U* = 576.00, *z* = 1.36 *p* = .175) and both are more “bluish” than that of *celeste.*

### Consensus in Naming BLUE Area by Modal Terms Celest*, Azzurr*, and Blu*

The employed unconstrained color-naming method implied that the elicited CTs were of considerable diversity; nevertheless, for each modal “blue” term, we found consensus higher than 50%. Percentage of the naming consensus for each chip (in Munsell coordinates) is presented in Tables S3–S5 of the Supplementary Materials. Among participants, consensus for each of the three “blue” terms is rather high for most of the chips. Consensus for blu* is higher than for both celest* and azzurr*, reaching 100% for chip 2.5PB 3/10; azzurr* consensus drops faster than that for celest*. Moreover, participants were rather unanimous (consensus >40%) in naming 8 chips (5B 6/8; 5B 7/8; 10B 5/8; 10B 6/6; 10B 6/10; 10B 7/8; 2.5PB 6/10; 2.5PB 7/8) using either celest* or azzurr* modal names.

Noteworthy, the chips with high(est) modal name consensus correspond to those frequently chosen as focal colors. Conversely, all (but one) chips chosen as focal colors reveal quite high modal name consensus rates: on average 44% (range: 16%–65%) for celest*, 40% (range: 29%–55%) for azzurr*, and 71% (range: 19%–100%) for blu*.

**Figure 9.**
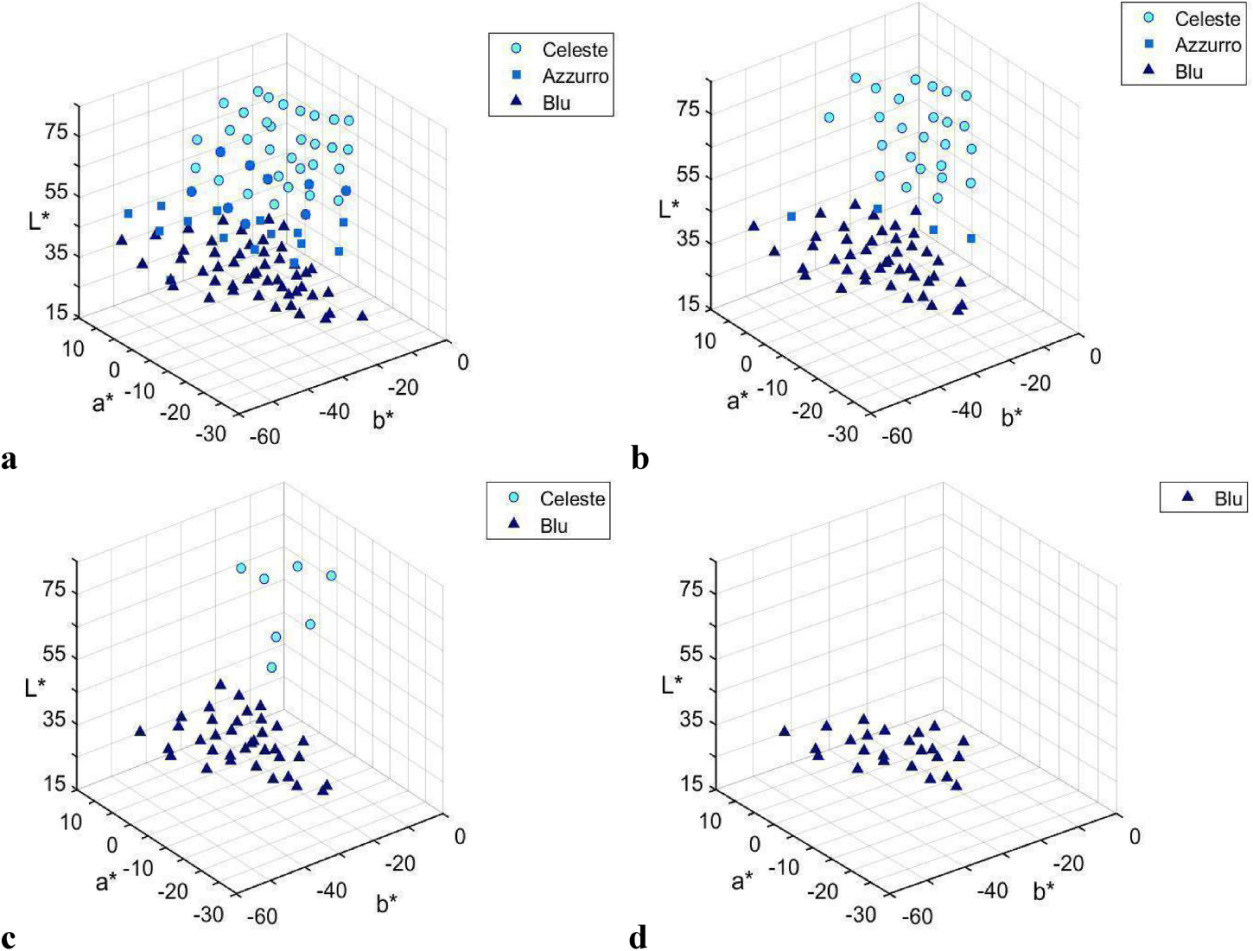
Coordinates (CIELAB space) of the chips with varying modal naming consensus: (a) 40%–49% (18, 21, and 7 chips for celest*, azzurr*, and blu*, respectively), (b) 50%–59% (18, 4, and 7 chips for celest*, azzurr*, and blu*, respectively), (c) 60%–69% (7 and 12 chips for celest* and blu*, respectively), and (d) 70% (24 chips for blu*).

Figure 9 shows the distribution of modal celest*, azzurr*, and blu* with consensus higher than 40%. It is apparent that for celest*, high consensus is observed for light blue shades of BLUE area, azzurr* for medium blue, and blu* for dark blue shades.

## Discussion

The results of the present study provide evidence that the Italian language, spoken in Tuscany, requires more than one BCT for naming BLUE area. The three terms, *celeste*, *azzurro*, and *blu*, appear to be deeply entrenched and indispensable for communicative efficiency for Tuscan speakers. To support our conclusion that *celeste*, *azzurro* and *blu* meet the criteria to be considered BCTs, we discuss our outcomes in relation to the two Berlin and Kay’s (1969/1991) criteria of basicness critical for our case: the term is (b) not a hyponym of another term and (d) psychologically salient.

The three terms are psychologically salient for all participants, according to the linguistic index—the occurrence in the inventories of all participants (apart from one who did not use *celeste*). *Celeste* ranks higher than *azzurro*, which, in turn, ranks higher than *blu*. Frequencies of use their modal terms are comparable, confirming the finding of Uusküla (2014). It is also worth noting that the Tuscan speakers’ inventory includes a great variety of elaborated terms associated with both *celeste* and *azzurro*—derivatives, compounded and suffixed words (see Table 1), that is, the means of codifying different nuances of hue, brightness, and saturation (cf., Grossmann & D’Achille, 2019). This contrasts with the inventories from two other locations in Italy: in Alghero, the elaborated “blue” terms were associated predominantly with *celeste* but with *azzurro* in Verona (Paramei et al., 2018, Table 1).

Furthermore, we consider denotative representations of the three “blue” terms with regard to stability of reference across participants, that is, one of the indices of psychological salience, criterion (d), and in relation to criterion (b), that the term should not be subsumed under the meaning of another term. We found that the three color categories are distinctly segregated along each dimension of CIELAB space (see Figures 4–8) suggesting that neither of these color categories is included in any other two BCCs. In particular, the central tendencies and dispersions of denotative distributions of the modal terms show no overlapping; neither the distributions of focal colors of the three “blue” terms overlap. Focal *azzurro* is intermediate in lightness straddling the gap between lighter *celeste* and darker *blu* foci.

Compared with centroids of focal colors of the three “blue” terms reported for Alghero and Verona samples (Paramei et al., 2018, Table 3), we observe that the mean of the Florence *celeste* focal (L* = 70.26, a* *=*  − 11.56, b* *=*  − 32.94) is similar to that for Alghero (L* = 63.93, a* *=*  − 11.07, b* *=*  − 39.58) In comparison, the Florence *azzurro* focal (L* = 61.87, a* *=*  − 8.12, b* *=*  − 40.77) is similar to that for Verona sample (L* = 68.80, a* *=*  − 9.58, b* *=*  − 33.58). The coordinates of *blu* focal are comparable for the three speaker samples, namely, Florence: L* = 30.80, a* = 5.71, b* =  − 45.25; Alghero: L* = 28.45, a* = 4.07, b* *=*  − 41.53; Verona: L* = 32.90, a * = 4.74, b* *=*  − 45.33. In addition, *azzurro* appears to be the “greenest” among the three terms, while both *azzurro* and *blu* are more “bluish” than *celeste*.

The consensus outcomes provide, too, an additional argument that Florence speakers require three BCTs to denote BLUE area. In particular, there is high consensus in using all three terms, with consensus for *blu* being the highest, which also is reflected in low variability among observers in their choices of focal *blu*.

Together, the obtained linguistic and psycholinguistic measures provide evidence, which justifies the conclusion that for Tuscan speakers all three main terms denoting the BLUE area—*celeste*, *azzurro*, and *blu*—perform as BCTs. Our findings are in accord with “persuasive rather than conclusive” results of the color-naming experiment carried out in Florence by Bimler and Uusküla (2014, p. A339) that Tuscan speakers differentiate *celeste*, denoting light blue, and *azzurro*, denoting medium blue, whereas *blu* denotes dark/navy blue, like in other regions in Italy. This is different from two BCTs, *azzurro* and *blu*, of Italian spoken in Veneto region and confirms the contention that Tuscan dialects have developed along their own lines; hence, their vocabulary does not always coincide with that of other regions (cf., Wieling et al., 2014).

By leaning upon “Linguistic historical background” (see the Addendum below), we conclude that the “triple Florence blues” probably resulted from the pressure for efficiency of communication (Jameson, & Komarova, 2009; Zaslavsky et al., 2019, 2022) vis-à-vis significant cultural practices in Middle-Ages Florence—flourishing of the blue dyeing technology and trade, and profuse employment of blue pigments in wall paintings and manuscripts. According to the latest findings on categorical perception, the linguistic “blue” distinction apparently enables greater chromatic precision of the conveyed blue-shade denotata (cf., Gibson et al., 2017) and facilitates the discrimination of colors because observers pay attention to the linguistic distinction between categories (cf., Witzel & Gegenfurtner, 2015).

In general, linguistic labels are argued to implicate the fine-grained memory of the percept corresponding to the category and as such, serve as the source of top-down information increasing efficacy of perceptual evidence integration—in color choice accuracy and in discrimination of cross-category members, compared to within-category members (Akbiyik et al., 2022).

### Addendum: Linguistic Historical Background of the “Triple Blues” in Florence, Tuscany

The process that led to speaking Standard Italian across the peninsula developed over the centuries. The Florentine dialect of the fourteenth century spread as the model for the Italian written language due to the cultural influence of three great Italian poets—Dante (*Divina Commedia*), Boccaccio (*Decameron*) and Petrarca (*Il Canzoniere*) (Migliorini, 1994). Florence also was the home of the *Accademia della Crusca* that set the standards for Italian language since 1582 and published in 1612 the first Dictionary of Italian language based on the Florentine dialect of the fourteenth century (Wieling et al., 2014).

In its phonology, the archaic Tuscan dialect (thirteenth and fourteenth centuries) retained a marked conservatism and was still close to Latin (De Mauro, 1983). In the texts of Dante and Boccaccio, the term *celeste* can be found with a slightly different spelling, *cilestro*, which is formed by analogy with *terrestre* “terrestrial” (Grossmann & D’Achille, 2016). *Azzurro* occurs twice in Dante’s and three times in Boccaccio’s texts (Grossmann & D’Achille, 2016).

Until the Italian unification (*Risorgimento*, 1815–1870) only 2% of the Italian population could speak proper Italian (De Mauro, 1983). Before the unification (1861), there were no forces capable of increasing linguistic homogeneity of various regions. The use of dialects was solid throughout the peninsula by popular strata, educated classes, aristocracies, and writers. Production of vernacular documents was more frequent in communities (e.g., Tuscany, Umbria, and Northern Italy), which were administered by bourgeoisie. Those institutions needed to let citizens know their dispositions and to communicate with homologous interlocutors (Castellani, 1952). In those early days, a common language was reserved only for those who had attended school (De Mauro, 1983).

The need for elementary education was established only with Napoleonic domination, forced by the bourgeoisie which, however, managed to achieve its goal only in northern Italian provinces, Piedmont and Lombardy−Veneto, in 1840. During the years of the unification, 0.8% of the population learnt Italian outside Rome and Tuscany (De Mauro, 1983).

As a result of the unification, in all regions of Italy the ruling class was formed which required a common language for effective communication. In addition, protective barriers between provinces were dismantled, and a unitary labor and capital market was created. Industrialization and urbanization have led to abandonment of dialects and adoption of a common tongue. Furthermore, industrialization made people move from rural regions to cities; as a result, the use of old local dialects was reduced.

De Mauro (1983) argues that regional varieties of Italian were the product of dialectophone efforts to learn Italian, whereby Italian people included elements of their own dialects in the common language, and the regional varieties acted as a link between the common language and the dialect, allowing a reciprocal exchange. The modern Standard Italian is quite a recent language, and the spread of the Florentine dialect as the common language has not eliminated the traditional multilingual aspect of Italy: no dialect has disappeared, and no minority language has become extinct (De Renzo, 2008).

The above brief overview on the history of Italian language is presented here for a better comprehension of why the number of BCTs to denote the BLUE area appeared to vary in different regions of Italy. For Florence specifically, it is conceivable that the need for linguistic elaboration of the BLUE area arose due to the development of complex woad dyeing technology in the woollen cloth industry, as attested by the Florentine treatise *Trattato d’Arte della Lana* (1419), which meticulously regulated qualities of dyed cloth (Cardon, 1992). The dyed samples, arranged from the darkest to the lightest shades of blue, significantly varied in their price and the corresponding names, among these *azurrini*, *cilestrini per Roma* (“for Roman taste”), *cilestrini al modo nostro* (“in the Florence fashion”).

In addition, lexical refinement of the BLUE area probably was encouraged by the fact that no later than from Middle Ages, different blue shades of artefacts were functioning as cultural signifiers (cf., Sahlins, 1976). In relation to the “triple blues” in Florence, it is helpful to recall Moss’s (1988) conjecture of the role of artistic culture, specifically of iconography, in stimulating linguistic refinement of the BLUE area. This is echoed by Gage (1999a, p. 109), who argues that “in literate societies, or those with a rich and detailed iconographic tradition, it may be possible to explore the particular significance of different colors in myths or rituals.”

In Italy, iconography played a significant role from the fifteenth century, when at least two different blue pigments, extracted from blue minerals, were used: ultramarine, obtained from by grinding high-quality lapis lazuli, was more brilliant, saturated and, hence, much more expensive than a pigment, “less pleasant … of azure earth-like colour,” obtained from azurite (Frison & Brun, 2016, p. 43). Therefore, in religious paintings, ultramarine was reserved for depicting the heavenly blue Virgin Mary’s mantle: “nothing suggests more that the religious colour-symbolism of the Renaissance must be subsumed under a more secular semiology of material value than this question of the Virgin’s robe, so often mentioned in Italian contracts in terms of the most expensive grade of ultramarine. The heavenly blue of the Virgin’s mantle has indeed seemed axiomatic to some twentieth-century commentators, just as it was to some of the later Middle Ages, for the access to understanding her nature offered by colour” (Gage, 1999b, p. 130).

In conclusion, we would like to reiterate Vidali’s (1983, p. 259) acumen: “what could be explained by the fact that, however a language codifies chromatic reality in terms of the spectrogram by assigning to “spaces” different labels varying from language to language, these labels tend to be the reflection of an experience determined by what it serves more to name, in terms of color, in a particular community” (authors' translation).

## Supplemental Material

sj-docx-1-ipe-10.1177_20416695221124964 - Supplemental material for Florence “blues” are clothed in triple basic terms Click here for additional data file. Supplemental material, sj-docx-1-ipe-10.1177_20416695221124964 for Florence “blues” are clothed in triple basic terms by Maria Michela Del Viva, Ilaria Mariani, Carmen De Caro and Galina V. Paramei in i-Perception

sj-docx-2-ipe-10.1177_20416695221124964 - Supplemental material for Florence “blues” are clothed in triple basic terms Click here for additional data file. Supplemental material, sj-docx-2-ipe-10.1177_20416695221124964 for Florence “blues” are clothed in triple basic terms by Maria Michela Del Viva, Ilaria Mariani, Carmen De Caro and Galina V. Paramei in i-Perception

sj-docx-3-ipe-10.1177_20416695221124964 - Supplemental material for Florence “blues” are clothed in triple basic terms Click here for additional data file. Supplemental material, sj-docx-3-ipe-10.1177_20416695221124964 for Florence “blues” are clothed in triple basic terms by Maria Michela Del Viva, Ilaria Mariani, Carmen De Caro and Galina V. Paramei in i-Perception

sj-docx-4-ipe-10.1177_20416695221124964 - Supplemental material for Florence “blues” are clothed in triple basic terms Click here for additional data file. Supplemental material, sj-docx-4-ipe-10.1177_20416695221124964 for Florence “blues” are clothed in triple basic terms by Maria Michela Del Viva, Ilaria Mariani, Carmen De Caro and Galina V. Paramei in i-Perception

sj-docx-5-ipe-10.1177_20416695221124964 - Supplemental material for Florence “blues” are clothed in triple basic terms Click here for additional data file. Supplemental material, sj-docx-5-ipe-10.1177_20416695221124964 for Florence “blues” are clothed in triple basic terms by Maria Michela Del Viva, Ilaria Mariani, Carmen De Caro and Galina V. Paramei in i-Perception
