# Supplementary material for: Florence “blues” are clothed in triple basic terms
Source: Iperception. 2022 Oct 3;13(5):20416695221124964. doi: 10.1177/20416695221124964 (PMC9536111; doi:10.1177/20416695221124964)
Supplement: sj-docx-1-ipe-10.1177_20416695221124964 - Supplemental material for Florence “blues” are clothed in triple basic terms [file sj-docx-1-ipe-10.1177_20416695221124964.docx]

**Supplementary Materials**

**Table S1.** Demographic characteristics of Italian monolingual participants tested in Florence (N=31). Birthplace and residency of all participants are in Tuscany. Education is indicated as the degree (Bachelor, Master’s) and the discipline.

| CODE | SEX | AGE | BIRTHPLACE | RESIDENCY | EDUCATION |
| --- | --- | --- | --- | --- | --- |
| AB | F | 21 | Florence | Figline Valdarno | BSc Psychology |
| AG | M | 24 | Florence | Florence | BA Design |
| AP | F | 21 | Pescia | Massa e Cozzile | BSc Psychology |
| BF | F | 20 | Florence | Capannori | BSc Psychology |
| BG | F | 20 | Bagno a Ripoli | Florence | BSc Psychology |
| CB | F | 21 | Arezzo | Castiglion Fiorentino | BSc Psychology |
| CB2 | M | 25 | Florence | Montecatini | MSc Architecture |
| CG | F | 20 | Cecina | Castagneto Carducci | BSc Psychology |
| EB | F | 21 | Florence | Lastra a Signa | BSc Psychology |
| FL | M | 24 | Pescia | Montecatini | MSc Engineering |
| FL2 | F | 25 | Florence | Florence | MEd Primary Education |
| FP | M | 25 | Prato | Montecatini | MSc Economy |
| FR | M | 26 | Pescia | Montecatini | BSc Physical Education |
| JA | M | 22 | Arezzo | Arezzo | BSc Psychology |
| LC | F | 21 | Pescia | Pieve a Nievole | BSc Psychology |
| LC2 | M | 24 | Pescia | Montecatini | MA Law |
| LL | M | 24 | Pescia | Montecatini | BA Political Science |
| LQ | F | 25 | Campi Bisenzio | Florence | MA Literature |
| LV | F | 21 | Arezzo | Arezzo | BSc Psychology |
| MB | M | 22 | Barga | Castel Nuovo di Garfagnana | BSc Psychology |
| MG | M | 24 | Florence | Montecatini | MSc Veterinary |
| MG2 | M | 25 | Fiesole | Florence | MA Law |
| MI | M | 26 | Florence | Florence | MA Law |
| MM | F | 19 | Florence | Florence | BSc Physical Education |
| MM2 | F | 22 | Florence | Bagno a Ripoli | BSc Communication Science |
| NG | F | 21 | Florence | Florence | BSc Psychology |
| NM | M | 25 | Pescia | Montecatini | BSc Economy |
| PN | M | 25 | Florence | Florence | MA Law |
| SB | F | 25 | Pisa | Vicopisano | MSc Psychology |
| SG | F | 21 | Arezzo | Arezzo | BSc Psychology |
| VM | F | 25 | Grosseto | Florence | MA Law |
