# Supplementary figures and images for: Florence “blues” are clothed in triple basic terms
Source: Iperception. 2022 Oct 3;13(5):20416695221124964. doi: 10.1177/20416695221124964 (PMC9536111; doi:10.1177/20416695221124964)

**a**
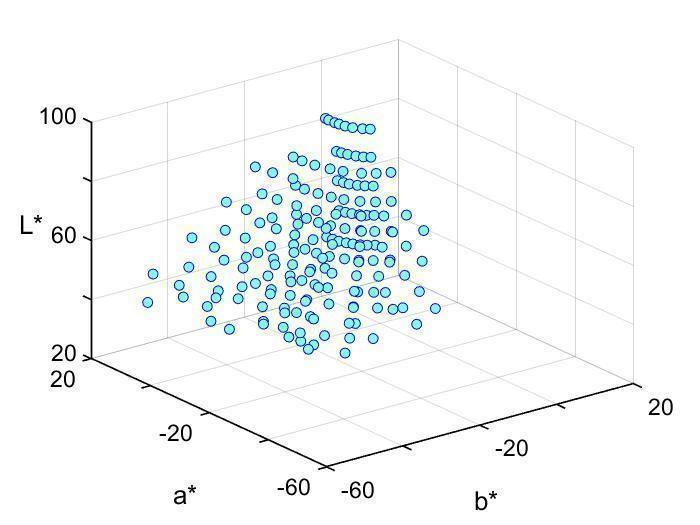


**b**
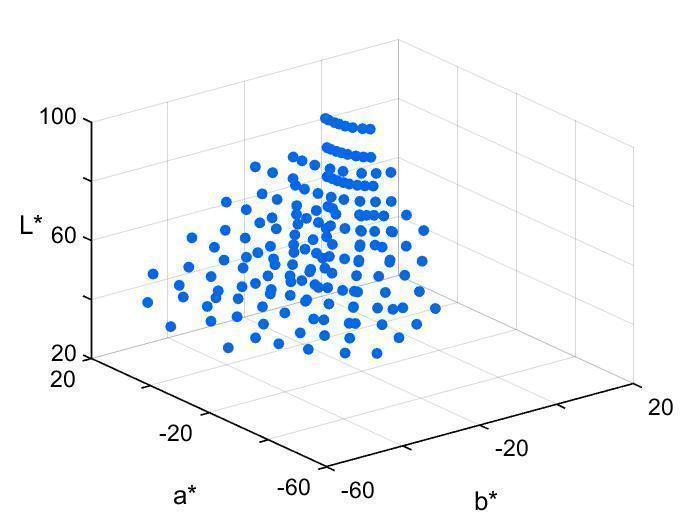


**c**
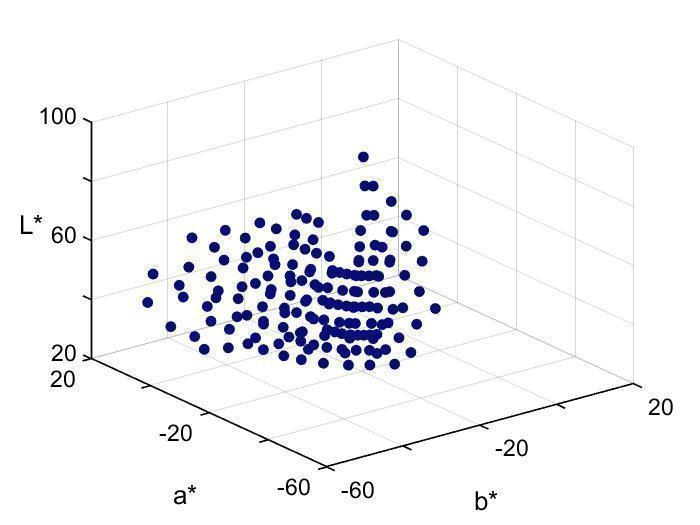


**Figure S1**. Distribution of denotata of modal terms (a) *celest**, (b) *azzurr** and (c) *blu** in CIELAB space*.*

Supplement: sj-docx-2-ipe-10.1177_20416695221124964 - Supplemental material for Florence “blues” are clothed in triple basic terms [file sj-docx-2-ipe-10.1177_20416695221124964.docx]
