# Supplementary material for: Florence “blues” are clothed in triple basic terms
Source: Iperception. 2022 Oct 3;13(5):20416695221124964. doi: 10.1177/20416695221124964 (PMC9536111; doi:10.1177/20416695221124964)
Supplement: sj-docx-3-ipe-10.1177_20416695221124964 - Supplemental material for Florence “blues” are clothed in triple basic terms [file sj-docx-3-ipe-10.1177_20416695221124964.docx]

**Table S3.** Consensus (%) for modal *celest** for each Munsell chart and individual chip. Consensus higher than 30% is highlighted as follows:

| 30-39% | 40-49% | 50-59% | 60-69% | 70-100% |
| --- | --- | --- | --- | --- |

| **7.5BG** |  | **2** | **4** | **6** | **8** | **10** |  | **7.5B** |  | **2** | **4** | **6** | **8** | **10** |  |
| --- | --- | --- | --- | --- | --- | --- | --- | --- | --- | --- | --- | --- | --- | --- | --- |
|  | **9** | 42 | 0 | 0 | 0 | 0 |  |  | **9** | 39 | 0 | 0 | 0 | 0 |  |
|  | **8** | 29 | 23 | 0 | 0 | 0 |  |  | **8** | 19 | 65 | 0 | 0 | 0 |  |
|  | **7** | 6 | 23 | 29 | 16 | 0 |  |  | **7** | 3 | 52 | 55 | 61 | 0 |  |
|  | **6** | 6 | 6 | 26 | 23 | 0 |  |  | **6** | 6 | 45 | 52 | 61 | 39 |  |
|  | **5** | 6 | 6 | 10 | 10 | 10 |  |  | **5** | 3 | 26 | 32 | 32 | 16 |  |
|  | **4** | 0 | 0 | 3 | 6 | 0 |  |  | **4** | 0 | 3 | 10 | 3 | 10 |  |
|  | **3** | 0 | 0 | 0 | 0 | 0 |  |  | **3** | 0 | 0 | 0 | 0 | 0 |  |
|  | **2** | 0 | 0 | 0 | 0 | 0 |  |  | **2** | 0 | 0 | 0 | 0 | 0 |  |
| **10BG** |  | **2** | **4** | **6** | **8** | **10** |  | **10B** |  | **2** | **4** | **6** | **8** | **10** | **12** |
|  | **9** | 48 | 0 | 0 | 0 | 0 |  |  | **9** | 19 | 0 | 0 | 0 | 0 | 0 |
|  | **8** | 39 | 45 | 0 | 0 | 0 |  |  | **8** | 16 | 52 | 61 | 0 | 0 | 0 |
|  | **7** | 10 | 48 | 35 | 23 | 0 |  |  | **7** | 3 | 39 | 58 | 52 | 0 | 0 |
|  | **6** | 6 | 32 | 32 | 19 | 0 |  |  | **6** | 6 | 35 | 42 | 58 | 45 | 0 |
|  | **5** | 3 | 16 | 19 | 6 | 3 |  |  | **5** | 3 | 29 | 35 | 42 | 23 | 16 |
|  | **4** | 0 | 6 | 3 | 0 | 0 |  |  | **4** | 0 | 13 | 10 | 6 | 0 | 0 |
|  | **3** | 0 | 3 | 3 | 3 | 0 |  |  | **3** | 0 | 6 | 6 | 0 | 0 | 0 |
|  | **2** | 0 | 0 | 0 | 0 | 0 |  |  | **2** | 0 | 3 | 0 | 0 | 0 | 0 |
| **2.5B** |  | **2** | **4** | **6** | **8** | **10** |  | **2.5PB** |  | **2** | **4** | **6** | **8** | **10** | **12** |
|  | **9** | 45 | 3 | 0 | 0 | 0 |  |  | **9** | 16 | 0 | 0 | 0 | 0 | 0 |
|  | **8** | 29 | 61 | 3 | 0 | 0 |  |  | **8** | 0 | 45 | 65 | 0 | 0 | 0 |
|  | **7** | 19 | 58 | 58 | 42 | 0 |  |  | **7** | 0 | 48 | 58 | 45 | 0 | 0 |
|  | **6** | 6 | 35 | 52 | 39 | 3 |  |  | **6** | 0 | 32 | 39 | 45 | 48 | 0 |
|  | **5** | 3 | 26 | 39 | 23 | 13 |  |  | **5** | 3 | 16 | 29 | 29 | 29 | 16 |
|  | **4** | 0 | 3 | 23 | 16 | 6 |  |  | **4** | 0 | 6 | 10 | 16 | 3 | 0 |
|  | **3** | 0 | 3 | 3 | 6 | 0 |  |  | **3** | 0 | 3 | 3 | 3 | 0 | 0 |
|  | **2** | 0 | 0 | 0 | 3 | 0 |  |  | **2** | 0 | 3 | 0 | 0 | 0 | 0 |
| **5B** |  | **2** | **4** | **6** | **8** | **10** |  | **5PB** |  | **2** | **4** | **6** | **8** | **10** | **12** |
|  | **9** | 39 | 0 | 0 | 0 | 0 |  |  | **9** | 3 | 0 | 0 | 0 | 0 | 0 |
|  | **8** | 19 | 58 | 0 | 0 | 0 |  |  | **8** | 0 | 45 | 58 | 0 | 0 | 0 |
|  | **7** | 3 | 52 | 61 | 55 | 0 |  |  | **7** | 0 | 29 | 48 | 52 | 0 | 0 |
|  | **6** | 3 | 32 | 55 | 52 | 32 |  |  | **6** | 0 | 13 | 39 | 45 | 35 | 0 |
|  | **5** | 3 | 23 | 35 | 23 | 26 |  |  | **5** | 3 | 13 | 29 | 32 | 29 | 19 |
|  | **4** | 0 | 0 | 13 | 6 | 13 |  |  | **4** | 3 | 6 | 6 | 13 | 16 | 10 |
|  | **3** | 0 | 0 | 3 | 3 | 0 |  |  | **3** | 0 | 3 | 0 | 3 | 0 | 0 |
|  | **2** | 0 | 0 | 0 | 0 | 0 |  |  | **2** | 0 | 3 | 0 | 0 | 0 | 0 |
