# Supplementary material for: Florence “blues” are clothed in triple basic terms
Source: Iperception. 2022 Oct 3;13(5):20416695221124964. doi: 10.1177/20416695221124964 (PMC9536111; doi:10.1177/20416695221124964)
Supplement: sj-docx-4-ipe-10.1177_20416695221124964 - Supplemental material for Florence “blues” are clothed in triple basic terms [file sj-docx-4-ipe-10.1177_20416695221124964.docx]

**Table S**4 Consensus (%) for modal *azzurr** for each Munsell chart and individual chip. Consensus higher than 30% is highlighted as follows:

| 30-39% | 40-49% | 50-59% | 60-69% | 70-100% |
| --- | --- | --- | --- | --- |

| **7.5BG** |  | **2** | **4** | **6** | **8** | **10** |  | **7.5B** |  | **2** | **4** | **6** | **8** | **10** |  |
| --- | --- | --- | --- | --- | --- | --- | --- | --- | --- | --- | --- | --- | --- | --- | --- |
|  | **9** | 39 | 0 | 0 | 0 | 0 |  |  | **9** | 39 | 0 | 0 | 0 | 0 |  |
|  | **8** | 26 | 26 | 0 | 0 | 0 |  |  | **8** | 16 | 35 | 0 | 0 | 0 |  |
|  | **7** | 16 | 29 | 23 | 16 | 0 |  |  | **7** | 3 | 35 | 39 | 35 | 0 |  |
|  | **6** | 6 | 26 | 26 | 23 | 0 |  |  | **6** | 0 | 35 | 39 | 32 | 42 |  |
|  | **5** | 6 | 13 | 19 | 19 | 16 |  |  | **5** | 0 | 16 | 35 | 45 | 42 |  |
|  | **4** | 0 | 6 | 10 | 3 | 0 |  |  | **4** | 0 | 13 | 19 | 19 | 19 |  |
|  | **3** | 0 | 0 | 3 | 0 | 0 |  |  | **3** | 0 | 3 | 0 | 6 | 0 |  |
|  | **2** | 0 | 0 | 0 | 0 | 0 |  |  | **2** | 0 | 0 | 0 | 0 | 0 |  |
| **10BG** |  | **2** | **4** | **6** | **8** | **10** |  | **10B** |  | **2** | **4** | **6** | **8** | **10** | **12** |
|  | **9** | 35 | 0 | 0 | 0 | 0 |  |  | **9** | 19 | 0 | 0 | 0 | 0 | 0 |
|  | **8** | 19 | 29 | 0 | 0 | 0 |  |  | **8** | 13 | 35 | 39 | 0 | 0 | 0 |
|  | **7** | 19 | 29 | 35 | 35 | 0 |  |  | **7** | 10 | 39 | 39 | 45 | 0 | 0 |
|  | **6** | 3 | 23 | 29 | 35 | 0 |  |  | **6** | 0 | 29 | 48 | 35 | 42 | 0 |
|  | **5** | 0 | 3 | 13 | 29 | 23 |  |  | **5** | 0 | 29 | 26 | 35 | 52 | 48 |
|  | **4** | 0 | 0 | 6 | 19 | 0 |  |  | **4** | 0 | 13 | 13 | 19 | 23 | 0 |
|  | **3** | 0 | 3 | 3 | 6 | 0 |  |  | **3** | 0 | 0 | 0 | 6 | 13 | 0 |
|  | **2** | 0 | 0 | 0 | 0 | 0 |  |  | **2** | 0 | 0 | 0 | 0 | 0 | 0 |
| **2.5B** |  | **2** | **4** | **6** | **8** | **10** |  | **2.5PB** |  | **2** | **4** | **6** | **8** | **10** | **12** |
|  | **9** | 39 | 3 | 0 | 0 | 0 |  |  | **9** | 10 | 0 | 0 | 0 | 0 | 0 |
|  | **8** | 19 | 35 | 3 | 6 | 0 |  |  | **8** | 6 | 39 | 35 | 0 | 0 | 0 |
|  | **7** | 13 | 32 | 39 | 42 | 0 |  |  | **7** | 3 | 32 | 29 | 42 | 0 | 0 |
|  | **6** | 6 | 39 | 35 | 42 | 0 |  |  | **6** | 3 | 16 | 35 | 39 | 42 | 0 |
|  | **5** | 0 | 19 | 32 | 52 | 48 |  |  | **5** | 0 | 19 | 29 | 45 | 48 | 55 |
|  | **4** | 0 | 10 | 16 | 32 | 3 |  |  | **4** | 0 | 3 | 10 | 13 | 26 | 0 |
|  | **3** | 0 | 3 | 6 | 10 | 0 |  |  | **3** | 0 | 0 | 0 | 0 | 0 | 0 |
|  | **2** | 0 | 0 | 0 | 0 | 0 |  |  | **2** | 0 | 0 | 0 | 0 | 0 | 0 |
| **5B** |  | **2** | **4** | **6** | **8** | **10** |  | **5PB** |  | **2** | **4** | **6** | **8** | **10** | **12** |
|  | **9** | 45 | 0 | 0 | 0 | 0 |  |  | **9** | 3 | 0 | 0 | 0 | 0 | 0 |
|  | **8** | 19 | 35 | 0 | 0 | 0 |  |  | **8** | 3 | 16 | 26 | 0 | 0 | 0 |
|  | **7** | 3 | 35 | 29 | 42 | 0 |  |  | **7** | 3 | 23 | 23 | 32 | 0 | 0 |
|  | **6** | 3 | 39 | 29 | 42 | 45 |  |  | **6** | 3 | 19 | 23 | 35 | 39 | 0 |
|  | **5** | 0 | 32 | 23 | 55 | 42 |  |  | **5** | 0 | 10 | 13 | 35 | 45 | 45 |
|  | **4** | 0 | 16 | 29 | 35 | 32 |  |  | **4** | 0 | 6 | 16 | 13 | 32 | 29 |
|  | **3** | 0 | 0 | 3 | 10 | 0 |  |  | **3** | 0 | 0 | 0 | 3 | 6 | 3 |
|  | **2** | 0 | 0 | 0 | 0 | 0 |  |  | **2** | 0 | 0 | 0 | 0 | 0 | 0 |
